# Risk Factors for Road-Traffic Injuries Associated with E-Bike: Case-Control and Case-Crossover Study

**DOI:** 10.3390/ijerph19095186

**Published:** 2022-04-24

**Authors:** Zhaohao Zhong, Zeting Lin, Liping Li, Xinjia Wang

**Affiliations:** 1 Injury Prevention Research Center, Shantou University Medical College, Shantou 515041, China; 20zhzhong@stu.edu.cn (Z.Z.); 18ztlin@stu.edu.cn (Z.L.); 2 School of Public Health, Shantou University, Shantou 515041, China; 3 The Second Affiliated Hospital of Shantou University Medical College, Shantou 515000, China; xj.wang2000@163.com; 4 Department of Orthopedic, Affiliated Cancer Hospital, Shantou University Medical College, Shantou 515041, China

**Keywords:** electric bicycle, road-traffic injury, case-control study, case-crossover study, risk factors

## Abstract

The Electric Bike (EB) has become an ideal mode of transportation because of its simple operation, convenience, and because it is time saving, economical and environmentally friendly. However, electric bicycle road-traffic injuries (ERTIs) have become a road-traffic safety problem that needs to be solved urgently, bringing a huge burden to public health. In order to provide basic data and a theoretical basis for the prevention and control of ERTIs in Shantou, mixed research combining a case-control study and a case-crossover study was carried out to investigate the cycling behavior characteristics and injury status of EB riders in Shantou city, and to explore the influencing factors of ERTI. The case-control study selected the orthopedic inpatient departments of three general hospitals in Shantou. The case-crossover study was designed to assess the effect of brief exposure on the occurrence of ERTIs, in which each orthopedic inpatient serves as his or her own control. Univariable and multivariable logistic regressions were used to examine the associated factors of ERTIs. In the case-control study, multivariable analysis showed that chasing or playing when cycling, finding the vehicle breakdown but continuing cycling, not wearing the helmet, and retrograde cycling were risk factors of ERTIs. Compared with urban road sections, suburb and township road sections were more likely to result in ERTIs. Astigmatism was the protective factor of ERTI. The case-crossover study showed that answering the phone or making a call and not wearing a helmet while cycling increased the risk of ERTIs. Cycling in the motor-vehicle lane and cycling on the sidewalk were both protective factors. Therefore, the traffic management department should effectively implement the policy about wearing a helmet while cycling, increasing the helmet-wearing rate of EB cyclists, and resolutely eliminate illegal behaviors such as violating traffic lights and using mobile phones while cycling. Mixed lanes were high-incidence road sections of ERTIs. It was suggested that adding people-non-motor-vehicles/motor vehicles diversion and isolation facilities in the future to ensure smooth roads and safety would maximize the social economic and public health benefits of EB.

## 1. Introduction

Road-traffic injuries (RTIs) are a major and urgent public health crisis in the world, and their incidence remains high in many countries [1,2,3]. According to the World Health Organization (WHO) Global Road Safety Report in 2018 [4], the global road-traffic accident death toll is as high as 1.35 million people per year, and more than half of them are vulnerable road users, such as pedestrians, cyclists, and motorcyclists. Road-traffic accidents are the leading cause of death for people aged 5–29, and the mortality rate has exceeded that of AIDS and tuberculosis [4], bringing huge economic losses to individuals and families, and even the entire country [5,6,7].

The Electric Bike (EB) has become an ideal mode of transportation because of its simple operation, and convenience, and because it is time saving, economical and environmentally friendly. In addition, in recent years, China has vigorously promoted low-carbon and green travel. In recent decades, EBs have gradually replaced motorcycles and bicycles as popular means of transportation [8]. Additionally, the public transportation service facilities in small and medium-sized cities are not as complete as those in large cities, which undoubtedly increases the proportion of EB in the transportation structure. 

According to statistics from the Ministry of Industry and Information Technology, EB production in China reached 27.077 million in 2019, and its social ownership is close to 300 million, ranking first in the world [9]. Electric bicycle road-traffic injuries (ERTIs) have become a road-traffic safety problem that needs to be solved urgently, bringing a huge burden to public health. ERTIs refer to accidents between EB and fixed objects, pedestrians, non-motorized (including bicycles, motorcycles), or motor vehicles (including cars, buses, trucks), and the incidents include crushing, scratching, overturning, explosion, fire, etc.

Current research mainly analyzes injury-monitoring data. Studies have shown that 27.6% of fatal bicycle accidents in the Netherlands involved EB in 2017 [10]; in Israel, a total of 3686 hospitalized cases were related to EB during 2014–2019 and 84.92% oral and maxillofacial injuries were attributed to EB [11]; and in the United States, there were 130,000 ERTI cases from 2000 to 2017, accounting for 5.3% of the total number of injuries in the emergency department. Among them, 17% of EB caused serious injuries such as traumatic brain injury and internal hemorrhage [12]; in Switzerland, the incidence of ERTI is 17%, and the main causes of injuries are road skidding, riding too fast, and being unable to maintain balance [13]. Langford used GPS technology to analyze the safety behaviors of EB cyclists in a complex traffic environment. The results showed that violations of traffic lights and driving on motorways are high-frequency dangerous riding behaviors [14]. Haustein et al. conducted a questionnaire survey on EB users in Denmark, and the results showed that the riding attitude of cyclists has a significant impact on heavy ERTIs [15]. After analyzing the accident data recorded by the Swiss police in 2011 and 2012, Weber et al. found that ERTI was dominated by Single-Bicycle Crashes (SBCs) [16]. 

The above-mentioned research mainly uses traffic-accident data, questionnaire surveys, and observation surveillance videos to study the characteristics and influencing factors of ERTIs, revealing the severity of ERTIs and the urgency of prevention. Due to the complex and diverse causes of RTIs, prevention and intervention should also be taken in multiple measures, and the effects of multiple factors on ERTIs should be comprehensively explored. For this reason, this study investigated risk factors for ERTIs in Shantou city. Therefore, the purpose of this study was to use a mixed research method of case-control study and case-crossover study to fully combine the characteristics of riders’ riding habits, road design, and environmental factors to analyze the relevant risk factors of ERTIs. This provides a scientific basis for preventing and reducing the occurrence of ERTIs, which has great practical significance.

## 2. Methods

### 2.1. Study Participants and Procedure

The case-control study selected the orthopedic inpatient departments of three general hospitals in Shantou (the First Affiliated Hospital of Shantou University, the Second Affiliated Hospital of Shantou University, and Shantou Central Hospital) for investigation. Nurses or physicians in the inpatient department conducted the investigation according to a uniform method. Including EB cyclists admitted to the hospital for ERTI into the case group. A 1:1 paired case-control study was adopted, and people of the same sex, same age (±5 years), same administrative region, with no ERTI in the 12 months before the survey were selected as the control group. The specific period before the injury of the same individual in the case group was defined as the hazard interval, and the three months from the day before the injury was defined as the control interval. A case-crossover study was conducted on the exposure of certain risk factors (such as helmet wearing, mobile phone use, etc.) in the two intervals.

### 2.2. Data Collection

#### 2.2.1. Case-Control Study

The orthopedic inpatient departments of three general hospitals in Shantou were selected for investigation. EB cyclists newly admitted to ERTI were included in the case group. After treatment, nurses or doctors in the inpatient department asked about the case uniformly and informed the research staff of the injury center. Researchers asked the injured EB cyclists about the situation and filled in the questionnaire of Characteristics of Electric Bicycle Usage and Road-Traffic Injuries in Shantou. Revised by injury epidemiology experts and improved after pre-investigation, the Cronbach’s coefficient was 0.781, which means that the data were reliable. In addition, the validity analysis was also analyzed. The KMO (Kaiser–Meyer–Olkin) value was 0.882; the Bart spherical value was 21,741.25, *p* < 0.001, indicating that the questionnaire had a good validity. The questionnaire mainly included three major contents: basic information, EB riding behaviors, and ERTIs characteristics. As follows: (1) Basic information: age, gender, education level, occupation, income, vision, etc.; (2) EB riding behaviors: including daily use (EB type, riding frequency, riding time, riding area, etc.) and dangerous behaviors (drunk driving, violating traffic lights, etc.); (3) ERTIs characteristics: including injury occurrence characteristics (time, location, injury type, road conditions, lighting conditions, weather, cause of injury, helmet and mobile phone use, etc.). Through 1:1 matching, EB cyclists of the same age (±5 years old), same gender, same administrative area, and no ERTI in the 12 months before the survey were selected as the control group.

#### 2.2.2. Case-Crossover Study

This was a technique for assessing the effect of brief exposure on the occurrence of rare acute outcomes [17], in which each person serves as his or her own control and can control some potential confounding factors such as age, sex, visual acuity, personality, etc. If the exposure was related to a rare event (or disease), then the frequency of exposure in the period just before the event should be higher than the frequency in the earlier period [17,18]. ERTI can be regarded as an emergency. The risk factors for the case-crossover study met the following conditions: (1) The case-crossover study explored the trigger or proximal cause of an acute event, which means the factor closest to the outcome of all causes; for example, an angry call received 5 min before the injury, a distraction one second before, etc. (2) The exposure changed rather than remaining stable throughout the study time. If the exposure itself was not short-lived, then its effect must have been short-lived, otherwise there would be no difference in exposure frequency between the hazard interval and the control interval. (3) Exposure factors had no carryover or cumulative effect, otherwise the distant past exposure might have been the cause. If the individual’s exposure could not be changed, then there was no basis for comparing the intervals of hazard and control. These factors include gender, occupation, age, etc. (4) The impact of exposure on individuals was always consistent, otherwise the effect on incentives could not be measured [19,20,21,22]. The objects of case-crossover study were divided into two parts, hazard interval and control interval, and the information was from the same individual. The hazard interval was defined as 15 min before the time of ERTI occurrence, and the control interval was defined as the daily frequency within 3 months before the occurrence of ERTI (from the day before the date of injury) [23]. Five questions were asked: “Did you wear a helmet at the time of the ERTI?”, “Did you wear headphones and play music at the time of the ERTI?”, “Did you answer the phone or make a call at the time of the ERTI?”, “Did you cycle on the motor vehicle lane at the time of ERTI?”, “Did you cycle on the sidewalk at the time of ERTI?”.

### 2.3. Statistical Analysis

EpiData 3.2 was used for double data entry verification. SPSS 25.0 (IBM, Armonk, NY, USA) was used for data analysis. Classification (categorical) data were described by frequency and percentage. Measurement (continuous) data were described by the mean and standard deviation (X ± SD). Comparisons of percentages were performed by the *χ*^2^ test or Fisher probabilities. The associated factors of ERTIs were examined using unconditional logistics regression analysis (maximum likelihood forward method), and the odds ratio (OR) and its 95% confidence interval (CI) were calculated. The explanatory variables were first analyzed by univariable analysis. Factors with statistical significance (*p* < 0.05) were entered into a model for multivariable analysis. The standard variable *α* was introduced as 0.05, and the standard variable excluded as *β* was 0.1. 

### 2.4. Ethics

This study was approved by the Ethics Committee of Shantou University Medical School (No. SUMC-2021-01) and we had also obtained the consent of participants. All participants gave their informed consent and volunteered to participate. 

## 3. Results

### 3.1. Case-Control Study

#### 3.1.1. Characteristics of Participants

A total of 151 severe ERTI cases from hospitals were collected in the study. According to the 1:1 matching principle, the investigation was randomly selected with the same sex, the same age (±5 years), the same administrative region and did not occur ERTI in the 12 months before the investigation as the control group. In the end, 142 cases and 142 controls were included. After testing, the difference in matching factors between the case group and the control group was not statistically significant, indicating that the two groups were balanced and comparable, as shown in Table 1.

#### 3.1.2. Analysis of Associated Factors of ERTIs

##### Univariable Analysis of Associated Factors of ERTIs

Univariable analysis was conducted on the participants, with significant differences found in finding a vehicle breakdown but continuing cycling; chasing or playing while cycling; myopia but not wearing glasses when cycling; drunk cycling; violating traffic lights; retrograde cycling; leaving the handlebar with one hand; frequently cycling in suburbs or townships (compared to urban areas); cycling while nervous; honking when cycling; average daily cycling time; cycling on the motor-vehicle lane; using mobile phones while cycling; tires skidding; risky behaviors such as shaking; almost colliding with other vehicles or pedestrians; placing heavy objects in the basket of the EB; brake failure; not wearing a helmet; myopia; and astigmatism (all *p* < 0.05) between ERTIs and non-ERTI. Finding a vehicle breakdown but continuing cycling; chasing or playing when cycling; and myopia but not wearing glasses when cycling were the top three dangerous riding behaviors, which increased the risk by 125.49, 100.41, and 50.47 times, respectively. Both myopia (OR = 0.39) and astigmatism (OR = 0.11) reduced the risk of ERTIs for riders, as shown in Table 2.

##### Multicollinearity Analysis

Tolerance (Tol) and variance inflation factor (VIF) were used to analyze the multicollinearity of variables significantly in univariable analysis before including them in multiple logistic regression. The maximum VIF was 4.265 and the corresponding Tol was 0.234. All the Tols were greater than 0.1 and the VIFs were less than 10, indicating that there is no multicollinearity between the variables. 

##### Multivariable Analysis of ERTIs

**Figure 1:**
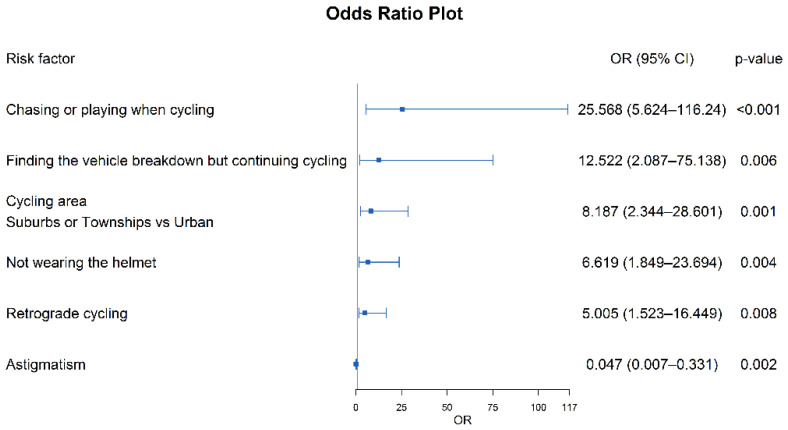
Forest plot of risk factors in the case-control study of ERTIs.

We finally performed a multivariable logistic regression analysis based on significant variables (*p* < 0.05) tested by univariable analysis together to estimate their ORs for ERTI. The explanatory variable assignment was shown in Table 3, and the results of all significant variables were displayed in Table 4 and Figure 1. Cyclists with astigmatism were less vulnerable to suffering from ERTIs (OR = 0.047, 95%CI: 0.007–0.331). Chasing or playing when cycling greatly increased the risk of ERTIs (OR = 25.57, 95%CI: 5.62–116.24). Compared with cycling in urban areas, cycling in suburbs or townships was more prone to ERTIs (OR = 8.19, 95%CI: 2.34–28.60). Cycling without a helmet had a higher risk of ERTIs (OR = 6.62, 95%CI: 1.85–23.70). In addition, finding a vehicle breakdown but continuing cycling (OR = 12.52, 95%CI: 2.09–75.14) and retrograde cycling (OR = 5.01, 95%CI: 1.52–16.45) were also important risk factors of ERTIs.

### 3.2. Case-Crossover Study

#### 3.2.1. Univariable Analysis of Associated Factors of ERTIs among Hazard Interval and Control Interval

The McNemar *χ*^2^ test was performed on the factors discussed in the case-crossover study. As shown in Table 5, the statistically significant factors were using a mobile phone while cycling, cycling on the motor-vehicle lane, and cycling on the sidewalk.

#### 3.2.2. Multivariable Logistic Regression Analysis of Associated Factors of ERTIs among Hazard Interval and Control Interval

**Figure 2:**
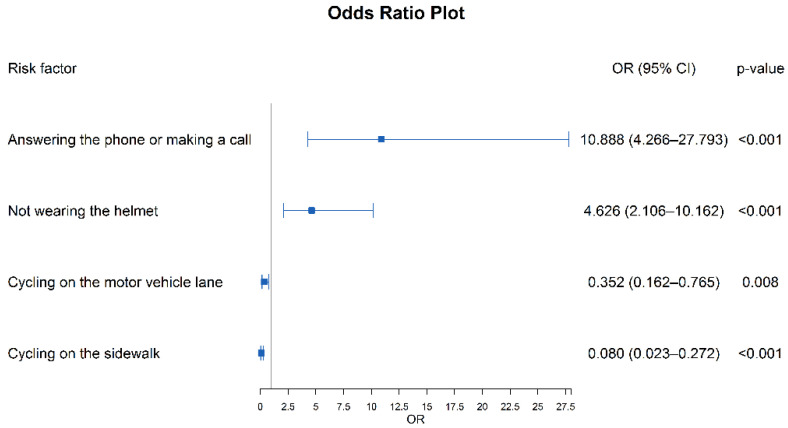
Forest plot of risk factors in the case-crossover study of ERTIs.

A paired logistic regression analysis was performed on the variables in Table 5, and the stepwise method of maximum likelihood estimation was adopted. Variables assignment was shown in Table 6. The statistically significant factors were shown in Table 7 and Figure 2. Wearing a helmet was a protective factor and the risk of injury while riding an electric bike without a helmet was 3.62 times higher than for those wearing helmets (OR = 4.626, 95%CI: 2.106–10.162). Using mobile phones while cycling (OR = 10.888, 95%CI: 4.266–27.793) increased the risk of injury by 9.89 times. Cycling on the motor-vehicle lane (OR = 0.353, 95%CI: 0.162–0.765) and sidewalk (OR = 0.008, 95%CI: 0.023–0.272) were both protective factors.

## 4. Discussion

Different from human-powered bicycles, EB were more attractive to pedestrians and car owners, especially in good weather conditions and traffic jams. Compared to traditional bicycles, EB were more convenient and effort-saving. Therefore, the explosive growth of EB led to intensified conflicts with motor vehicles. It also contributed to the increase in the incidence of ERTI [24]. 

A number of studies confirmed that many EB cyclists lack road-traffic safety knowledge and have weak awareness of laws and traffic-safety regulations [25,26]. This was also the reason for the endless emergence of various illegal and dangerous riding behaviors of EB cyclists. The incidence of dangerous behaviors such as “drunk cycling”, “retrograde cycling”, “violating traffic lights”, and “using mobile phones while riding” among people with ERTIs was higher than that of non-ERTIs. The result was also supported by previous studies [27]. Studies had also shown that chasing or playing while cycling was a risk factor leading to ERTIs, and wearing a safety helmet was a proven and effective preventive measure [18,28]. That was consistent with the results we obtained through the case-control study and case-crossover study. Previous studies showed that using a mobile phone while cycling will increase the possibility of injuries [29,30], and wearing a high-quality helmet can reduce the risk of serious injury by 70.00% and the risk of death from road-traffic injuries by 40.00% [4]. A large number of studies confirmed that wearing a safety helmet was an effective measure to prevent ERTIs. When an injury occurs, the safety helmet can absorb most of the impact force, avoid shocking the head, and greatly reduce the severity of injury and mortality of ERTI [31]. Therefore, it was necessary to increase the correction and intervention of such dangerous behaviors in the future. Astigmatism was one of the protective factors for ERTIs in this study. This may be related to the fact that astigmatism sufferers were limited by their vision, so they were more cautious about road conditions, and rode slower. It may also be due to the uneven distribution of astigmatism among the participants. Non-astigmatism accounted for 60% of participants, and the overall astigmatism rate was low, which may lead to bias in results. Therefore, it needs to be further studied in the future. 

Using a mobile phone to answer the phone or make a call while riding EB increased the risk of ERTI by approximately 10 times, which was similar to the results of previous studies [27,32,33]. It can cause visual, behavioral, and cognitive distractions, neglecting to observe the surrounding environment. The research of Haque et al. [34,35] found that making or receiving phone calls diverted the driver’s attention from driving conditions, prolonged the response time to surrounding dangers, and weakened emergency response skills. The South Korean government had invested in a mobile phone application that can automatically lock the phone screen after walking more than seven steps, and unlock it when pedestrians stop walking completely [18]. However, the implementation of this regulation caused great controversy, and its actual operability was also open to debate. Therefore, this study suggested that the most important intervention at the present stage was to strengthen government propaganda and media attention to make people aware of the potential dangers and serious consequences of using mobile phones while cycling. 

Drunk cycling was an important risk factor for ERTIs [36]. The results of this study showed that drunk cycling increased the risk of ERTI by about 27 times. Cycling under the influence of alcohol, the judgment of the road environment, the control of the vehicle, the perception of danger, and the riding ability were all reduced, which was easy to lead to ERTI. In addition, drunk cycling can also cause a series of dangerous behaviors such as speeding, violating traffic laws, and risky behaviors such as shaking and leaving the handlebar with one hand, which further increase the risk of injury [37]. EB, as a non-motor vehicle, rode at a much slower speed than motor vehicles. Many cyclists were prone to fluke and thought that it is harmless to keep cycling at a low speed after drinking, and the current laws and regulations related to drunk cycling are still inadequate and imperfect. It was also difficult for the road-traffic department to supervise cyclists riding under the influence of alcohol. In terms of prevention of ERTI, it was necessary to focus on strengthening safety education about the dangers of drunk cycling and promulgating relevant laws and regulations to strictly prohibit cycling under the influence of alcohol.

Cycling on motor vehicles and sidewalks were both protective factors found in the case-crossover study, which was different from previous studies [38,39]. Previous investigations found that about 3% of EBs have the behavior of riding on motorways [14,26,40], and this behavior increased the risk of ERTI by 60% [39]. Relying on their dexterity and lightness, EBs can arbitrarily ride into the space in the mixed lanes, and even change their riding direction. This behavior often made faster motor vehicles too late to avoid, which led to injury accidents. At the same time, there are many types of vehicles on mixed lanes and drive at different speeds. It was suggested that road facilities should add people-non-motor vehicles-motor vehicles diversion and isolation facilities in the future to ensure smooth roads and safety. The well-planned motor lanes had better traffic orders. 

Retrograde cycling was an important risk factor for ERTIs. Retrograde cycling is a serious road-traffic violation. It not only disrupts road-traffic order, reduces road-passing capacity, and causes road-traffic system congestion, but also further increases the risk of collisions with other road-traffic participants, leading to accidents. Compared with cars, EBs were smaller, more dexterous, and made retrograde cycling easier. Road-traffic departments also lacked the construction of electronic monitoring and credit-deduction systems for such vehicles in retrograde. The cost of violations was lower. 

Our research also revealed that cycling in suburbs or townships was at higher risk for ERTIs compared to urban areas, which was aligned with previous studies [41,42]. This may be since the roads in rural areas are more rugged and the road-traffic construction is not perfect, so accidents are more likely to occur. Therefore, more attention should be paid to the surrounding road conditions when cycling in rural areas. This also reminds us that road construction in rural areas needs to be improved by repairing rugged roads and building warning signs in areas with high accident rates.

In addition, we should ensure the safety of the EB. It is suggested that we should not take risks in some situations of EB cycling such as brake failure, tires skidding, and others, faults to prevent ERTIs. Additionally, heavy objects should not be placed in the basket of EB, so as not to affect the cycling.

In this study, a mixed research method combining the case-control study and case-crossover study was carried out to investigate ERTIs, which clearly provided a comprehensive perspective and scientific data support for the effective prevention of ERTIs.

The limitations of this study mainly include the following: firstly, only three comprehensive hospitals in Shantou were selected as investigation points for injury cases, and all three hospitals were located in the downtown area of Shantou, which limited the extrapolation of the research results to a certain extent. Secondly, the exposure factors of ERTIs were the key information of this study. However, due to the low degree of some ERTIs, the respondents may have unavoidable recall bias when self-filling the questionnaire. Thirdly, the traditional case-control study was one of the most basic and important research types in analytical epidemiological methods. It can widely screen suspicious risk factors. Meanwhile, the case-crossover study served as a derivative of case-control studies, which was more suitable for acute events such as ERTIs inducement or proximate cause. Therefore, the risk factors revealed by two methods used in this study at the same time were slightly different. This study provided clues for further research, which needed to be validated by future prospective studies.

## 5. Conclusions

Our focus for ERTIs preventive interventions should target retrograde cycling, violating traffic lights, and drunk cycling. Mixed lanes are high-risk roads for ERTIs. ERTIs are more likely to occur in suburban and township sections than in urban sections. Wearing a helmet can reduce the risk of ERTI by about five times. In the future, traffic-management departments should improve the registration and management system of licenses of EB and the policy of mandatory helmet wearing. In addition, traffic-safety promotion could focus on publicizing the hazards of using mobile phones during cycling at main traffic intersections or landmarks and strengthen the road-traffic safety awareness of cyclists by coordinating with multiple departments for road planning and infrastructure construction, using the big data platform to realize the joint construction and sharing of injury information, dynamically monitoring the occurrence of ERTIs, and creating a safe traffic environment. Therefore, policymakers and administrators are supposed to take these associated factors into full consideration when formulating policies to prevent ERTIs, to create a safe traffic environment, and maximize the benefits of EBs.

## Figures and Tables

**Table 1:** Comparison of baseline conditions between the case group and the control group, *n* (%).

Variables	Case Group *n* = 142	Control Group *n* = 142	*χ*^2^/t	*p*-Value
Gender			<0.001	1.000
Male	71 (50.35)	71 (50.35)		
Female	71 (49.65)	71 (49.65)		
Age	36.16 ± 8.369	35.78 ± 6.098	−0.411	0.681
Areas			<0.001	1.000
Urban	141 (100.00)	141 (100.00)		
Rural				
Own EB			<0.001	1.000
Yes	68 (48.23)	68 (48.23)		
No	73 (51.77)	73 (51.77)		

**Table 2:** Univariable analysis of risk factors of EB.

Risk Factors	OR	OR 95%CI	*χ* ^2^	*p*-Value
Finding the vehicle breakdown but continuing cycling	126.490	30.034–532.729	127.082	<0.001
Chasing or playing while cycling	101.409	30.605–336.021	136.909	<0.001
Myopia but not wearing glasses when cycling	50.474	17.671–144.170	105.717	<0.001
Drunk cycling	34.840	15.123–80.264	110.348	<0.001
Violating traffic lights	24.846	13.074–47.216	121.431	<0.001
Retrograde cycling	20.583	11.214–37.782	114.946	<0.001
Leaving the handlebar with one hand	19.144	10.051–36.463	101.728	<0.001
Frequently cycling in suburbs or townships (compared to urban areas)	17.646	8.317–37.439	77.359	<0.001
Cycling while nervous	14.964	8.332–26.875	96.194	<0.001
Honking when cycling	11.346	6.223–20.687	74.067	<0.001
Average daily cycling time (compared to less than 10 min)			23.532	<0.001
More than 1 h	10.946	2.362–50.730		0.002
30 min–1 h	3.193	1.547–6.588		0.002
10–30 min	1.142	0.659–1.978		0.635
Cycling on the motor-vehicle lane	7.508	4.179–13.489	51.574	<0.001
Using mobile phones while cycling	7.418	4.374–12.582	60.123	<0.001
Tires skidding	3.814	2.321–6.267	29.009	<0.001
Risky behaviors such as shaking	3.428	1.822–6.449	15.625	<0.001
Almost colliding with other vehicles or pedestrians	3.064	1.879–4.995	20.717	<0.001
Placing heavy objects in the basket of the EB	2.821	1.672–4.760	15.592	<0.001
Brake failure	2.675	1.654–4.328	16.398	<0.001
Not wearing a helmet	2.500	1.328–4.717	8.388	0.004
Myopia	0.385	0.238–0.623	15.447	<0.001
Astigmatism	0.111	0.048–0.258	34.047	<0.001

**Table 3:** Variable assignment for the multivariable logistic regression analysis.

Variables	Assignment
Myopia	No = 0, Yes = 1
Astigmatism	No = 0, Yes = 1
Cycling area	Urban = 0, Suburbs or Townships = 1
Average daily cycling time	Less than 10 min = 0, 10–30 min = 1, 30 min–1 h = 2, More than 1 h = 3
Honking when cycling	No = 0, Yes = 1
Drunk cycling	No = 0, Yes = 1
Not wearing a helmet	No = 0, Yes = 1
Placing heavy objects in the basket of the EB	No = 0, Yes = 1
Cycling nervous	No = 0, Yes = 1
Tires skidding	No = 0, Yes = 1
Brake failure	No = 0, Yes = 1
Retrograde cycling	No = 0, Yes = 1
Violating traffic lights	No = 0, Yes = 1
Leaving the handlebar with one hand	No = 0, Yes = 1
Chasing or playing while cycling	No = 0, Yes = 1
Using mobile phones while cycling	No = 0, Yes = 1
Risky behaviors such as shaking	No = 0, Yes = 1
Finding a vehicle breakdown but continuing cycling	No = 0, Yes = 1
Cycling on the motor vehicle lane	No = 0, Yes = 1
Myopia but not wearing glasses when cycling	No = 0, Yes = 1

**Table 4:** Multivariable logistic regression analysis on case-control study of ERTIs.

Factors	β	*χ* ^2^	OR	OR 95%CI	*p*-Value
Chasing or playing when cycling	3.241	17.600	25.568	5.624–116.240	<0.001
Finding the vehicle breakdown but continuing cycling	2.527	7.643	12.522	2.087–75.138	0.006
Cycling area					
Suburbs or Townships vs. Urban	2.103	10.854	8.187	2.344–28.601	0.002
Not wearing a helmet	1.890	8.437	6.619	1.849–23.694	0.004
Retrograde cycling	1.610	7.038	5.005	1.523–16.449	0.008
Astigmatism	−3.061	9.418	0.047	0.007–0.331	0.002

**Table 5:** Univariable analysis of associated factors of ERTIs among hazard interval and control interval.

Variables	Hazard Interval	Control Interval	*χ* ^2^	*p*-Value
Wearing a helmet			0.231	0.631
Yes	95 (62.91)	99 (65.56)		
No	56 (37.09)	52 (34.44)		
Wearing headphones and playing music			0.577	0.447
Yes	47 (31.13)	41 (27.15)		
No	104 (68.87)	110 (72.85)		
Answering the phone or making a call			46.236	<0.001
Yes	109 (97.19)	50 (33.11)		
No	42 (27.81)	101 (66.89)		
Cycling on the motor vehicle lane			12.409	<0.001
Yes	31 (79.47)	59 (39.07)		
No	120 (20.53)	92 (60.93)		
Cycling on the sidewalk			29.307	<0.001
Yes	12 (7.95)	50 (33.11)		
No	139 (92.05)	101 (66.89)		

**Table 6:** Assignment of case-crossover study Variables.

Variables	Hazard Interval	Control Interval	Assignment
Not wearing a helmet	The occurrence of ERTIs	Within three months before the ERTI	No = 0, Yes = 1
Wearing headphones and playing music	The occurrence of ERTIs	Within three months before the ERTI	No = 0, Yes = 1
Answering the phone or making a call	15 min before the ERTIs occurred	Within three months before the ERTI	No = 0, Yes = 1
Cycling on the motor vehicle lane	The occurrence of ERTIs	Within three months before the ERTI	No = 0, Yes = 1
Cycling on the sidewalk	The occurrence of ERTIs	Within three months before the ERTI	No = 0, Yes = 1

**Table 7:** Multivariable logistic regression analysis on case-crossover study of ERTIs.

Risk Factors	β	*χ* ^2^	OR	OR 95%CI	*p*-Value
Answering the phone or making a call	2.388	24.938	10.888	4.266–27.793	<0.001
Not wearing the helmet	1.532	14.551	4.626	2.106–10.162	<0.001
Cycling on the motor vehicle lane	−1.046	6.948	0.352	0.162–0.765	0.008
Cycling on the sidewalk	−2.529	16.330	0.080	0.023–0.272	<0.001

## Data Availability

The datasets used and analyzed in the study are available from the corresponding author on reasonable request.

## Abbreviations

WHO—World Health Organization; OR—odds ratio; CI—confidence intervals; EB—electric bicycle; RTI—road-traffic injury; ERTI—electric bicycle road traffic injury; SBC—single-bicycle crash.
